# Supplementary material for: Identification of photoperiod-induced specific miRNAs in the adrenal glands of Sunite sheep (Ovis aries)
Source: Front Vet Sci. 2022 Jul 22;9:888207. doi: 10.3389/fvets.2022.888207 (PMC9354845; doi:10.3389/fvets.2022.888207)
Supplement: Supplementary file 1 [file Data_Sheet_1.ZIP › Supplementary file/miRDeep2 file.zip!/miRDeep2 file/Know_Structure/SP42A2/oar-mir-758.pdf]

miRBase precursor : oar-mir-758
Total read count : 556
oar-miR-758-5p read count : 0
oar-miR-758-3p read count : 552
remaining reads : 4

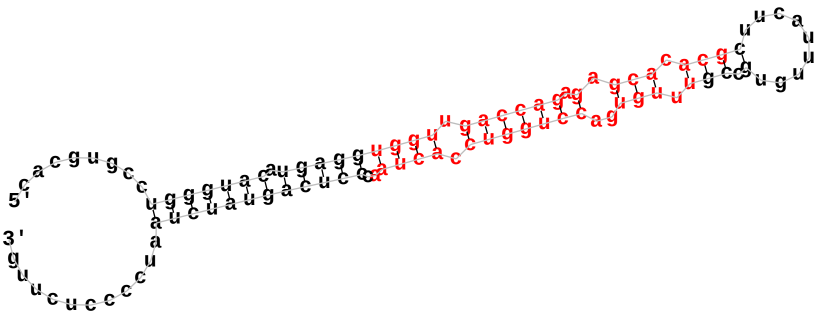

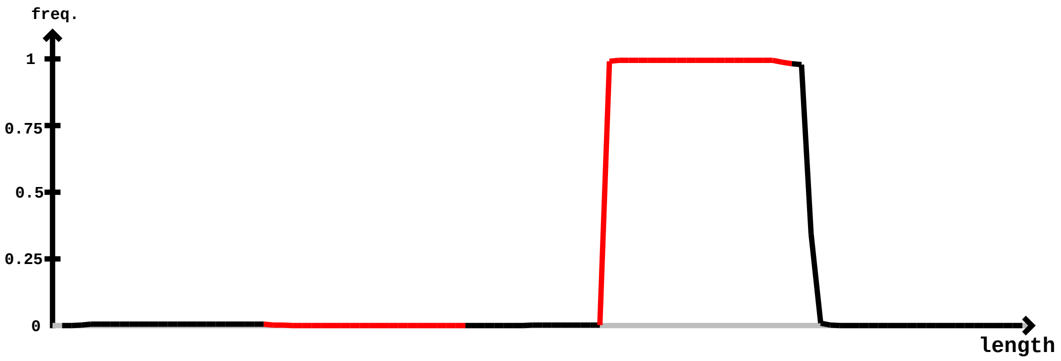


```
                                                         oar-miR-758-3p
                    oar-miR-758-5p
5'- cacgugccuggguacaugaggugguugaccagagagcacacgcuucauuugugccguuugugaccugguccacuaacccucaguaucuaauccccucuug   -3'   exp
    ........((((((.(((((((((.((((((.(.(((.((((.........).))).)))..))))))).)))).))))))))))).............   reads   mm   sample
    .acgugccuggguacaugagg...............................................................................   1       0    seq
    ..cgugccuggguacaugagg...............................................................................   1       0    seq
    ..cgugccuggguacaugaggug.............................................................................   1       0    seq
    ................................................uugugccguuugugaccugguccacu..........................   1       0    seq
    ........................................................uuugugaccugguccacu..........................   3       0    seq
    ........................................................uuugugaccugguccacua.........................   3       0    seq
    ........................................................uuugugaccugguccacuaa........................   2       0    seq
    ........................................................uuugugaccugguccacuaac.......................   352     0    seq
    ........................................................uuugugaccugguccacuaacc......................   185     0    seq
    ........................................................uuugugaccugguccacuaaccc.....................   4       0    seq
    ........................................................uuugugaccugguccacuaacccu....................   1       0    seq
    .........................................................uugugaccugguccacuaac.......................   1       0    seq
    .........................................................uugugaccugguccacuaacc......................   1       0    seq
```
